# MAP4K3 Is a Component of the TORC1 Signalling Complex that Modulates Cell Growth and Viability in *Drosophila melanogaster*

**DOI:** 10.1371/journal.pone.0014528

**Published:** 2011-01-18

**Authors:** Martín Resnik-Docampo, Jose F. de Celis

**Affiliations:** Centro de Biología Molecular Severo Ochoa, Consejo Superior de Investigaciones Científicas and Universidad Autónoma de Madrid, Madrid, Spain; Katholieke Universiteit Leuven, Belgium

## Abstract

**Background:**

MAP4K3 is a conserved Ser/Thr kinase that has being found in connection with several signalling pathways, including the Imd, EGFR, TORC1 and JNK modules, in different organisms and experimental assays. We have analyzed the consequences of changing the levels of MAP4K3 expression in the development of the *Drosophila* wing, a convenient model system to characterize gene function during epithelial development.

**Methodology and Principal Findings:**

Using loss-of-function mutants and over-expression conditions we find that MAP4K3 activity affects cell growth and viability in the Drosophila wing. These requirements are related to the modulation of the TORC1 and JNK signalling pathways, and are best detected when the larvae grow in a medium with low protein concentration (TORC1) or are exposed to irradiation (JNK). We also show that MAP4K3 display strong genetic interactions with different components of the InR/Tor signalling pathway, and can interact directly with the GTPases RagA and RagC and with the multi-domain kinase Tor.

**Conclusions and Significance:**

We suggest that MAP4K3 has two independent functions during wing development, one related to the activation of the JNK pathway in response to stress and other in the assembling or activation of the TORC1 complex, being critical to modulate cellular responses to changes in nutrient availability.

## Introduction

The *Drosophila* wing imaginal disc is a model system to study epithelial development, and is particularly well suited to analyse the involvement of signalling pathways in the regulation of organ growth and pattern formation [1]–[4]. The wing disc primordium is specified in the embryonic ectoderm as a group of approximately 30 cells that proliferate during the larval stages to form the mature disc [1]. The proliferative stage of the disc is accompanied by the specification of different territories, and by the commitment of cells to specific cell fates [2]. Most conserved signalling pathways have being shown to play determining roles in the control of cell proliferation, cell survival and during the specification of cell identities in the wing disc. Furthermore, genetic modifications in the activity of individual pathways result in very specific phenotypes in the wing, the adult structure that differentiates from the central region of the wing disc epithelium. These phenotypes can be used as a diagnostic to assign genes identified through genetic screens to individual signalling pathways [5], [6]. In addition, the proliferation and differentiation of wing cells is very sensitive to changes in the level of signalling, allowing the identification of additional components of these pathways by both loss- and gain-of-function genetic screens.

One of the pathways that play a prominent role in the regulation of cell growth is the Insulin receptor pathway (InR), which activity is required to promote cellular growth and progression through the cell cycle in response to growth factors [7]–[11]. In general, mutations that decrease Insulin signalling in the wing cause the formation of smaller than normal wings, due to a reduced number of cells, and more prominently, to a reduction in cell size. Insulin signalling is to a large extent mediated by the activation of the TORC1 complex [12], following a linear pathway that is well characterised in both vertebrates and invertebrates [13]–[16]. This pathway involves the inactivation of the GTPase activating protein TSC1/2 by Protein Kinase B (PKB/Akt)-mediated phosphorylation [17]–[19]. The inactivation of TSC1/2 allows the accumulation of the small GTPase Rheb in its GTP-bound form [20]–[23], and the assembly of a multiprotein complex containing the Ser/Thr kinase protein Tor (Target of Rapamicin) and the substrate adaptor protein Raptor, among other components. This complex, named TORC1 is able to activate ribosomal S6 kinase (S6K) [24]–[26] and to inactivate 4E-binding protein (4E-BP) [24], [27], an inhibitor of the eukaryotic initiation factor 4E (eIF-4E). These effects lead to ribosome complex formation and translation, and to the regulation of protein synthesis and cellular growth. In addition to Insulin signalling, the activity of TORC1 is also regulated by intracellular levels of aminoacids, using a parallel pathway that is independent of Rheb [28], [29]. This branch of the pathway confers to TORC1 the ability to act as a sensor for nutrients, and although it is less-well characterized, recent studies have implicated several aminoacid transporters [30], [31], the Ras-related proteins RagA, RagB, RagC and RagD [32]–[34], and MAP4K3 as upstream components of TORC1 [35], [36]. Thus, it appears that the nucleotide status of Rag proteins is regulated by amino acids and perhaps by MAP4K3, and when RagA/B dimmers are in a GTP-bound form and RagC/D dimmers in a GDP-bound form they can interact with and activate TORC1.

We isolated one P-UAS insertion in the 5′ region of CG7097 [5], a gene recently named *happyhour* (*hppy*) due to the increased resistance to ethanol-induced sedation displayed by hypomorphic alleles [37]. *hppy* encodes the *Drosophila* orthologue of MAP4K3, a Ser/Thr Kinase belonging to the Germinal Center Kinase I (GCK-I) family of Ste20-related kinases [38]. This protein is characterized by a Ser/Thr Kinase catalytic domain and by a Citron-homology domain that might be involved in protein-protein interactions. MAP4K3 has being implicated in different cellular processes based in biochemical and cellular assays carried out in different experimental systems. The proposed requirements of MAP4K3 include the regulation of the Imd pathway in response to *E. coli* infection in fly cells, the inhibition of EGFR signalling in fly brain neurons, the nutrient regulation of TORC1 signalling in fly and human cell cultures and the induction of the intrinsic cell death pathway [16], [35]–[37], [39]–[42]. More recently, a MAP4K3 mutant has being found to display phenotypes characteristic of low TORC1 activity [36]. We have used the advantages of the *Drosophila* wing model to analyze the functional requirements of *hppy/*MAP4K3 in epithelial development. We show that loss of MAP4K3 results in a weak reduction in cell size, and that this phenotype is very sensitive to nutrient availability. In addition, increased expression of MAP4K3 induces cell death and strongly down-regulates TORC1 signalling. The MAP4K3 protein is expressed in the cytoplasm of all wing disc cells, and pull-down experiments indicate that this protein can interact with Rag GTPases and Tor, suggesting that it is a component of the TORC1 complex. Cells expressing increased levels of MAP4K3 behave similarly to gain of Tor expression, suggesting that the over-expression of MAP4K3 disrupt protein complexes involving Tor.

## Results

### Increased expression of *hppy* reduces the size of the wing

**Figure 1:**
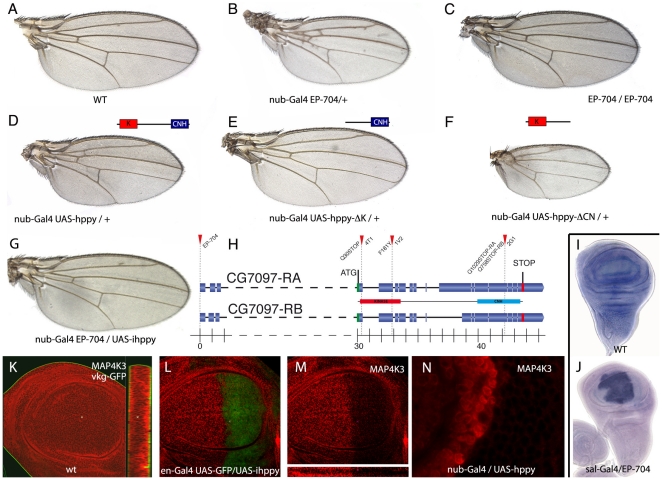
The phenotype of *EP-704/Gal4* combinations is caused by increased expression of MAP4K3/*happyhour*. (A) Wild type wing. (B) *nub-Gal4 EP-704*/+ wing. *nub-Gal4* is expressed in the wing blade and hinge during the second and third larval instars. (C) *EP-704* homozygous wing. This wing is reduced in size compared to wild type by a 12%. (D) *nub-Gal4 UAS-hppy/+* wing, showing the same phenotype of smaller than normal wing size than *nub-Gal4 EP-704*/+. (E) *nub-Gal4/UAS-hppy-ΔK* wing showing a wild type phenotype. (F) *nub-Gal4/UAS-hppy-ΔCN* wing showing a strong reduction in the size of the wing hinge and blade. (G) *EP-704 nub-Gal4/UAS-ihppy* wing, showing a rescue of the *nub-Gal4 EP-704/+* phenotype. (H) Gene structure of *hppy*, showing the two transcripts produced by alternative splicing (CG7097-RA and CG7097-RB), the position of the *EP-704* insertion in the first non-coding exon and the positions of the mutations found in the revertants *hppy^4T1^* (Q30Stop), *hppy^1V2^* (F181Y) and *hppy^2G1^* (Q758Stop). The Hppy protein (MAP4K3, shown below) contains a Ser/Thr kinase domain (red) and a Citron Homology domain (blue). The genomic coordinates are indicated below in Kb. (I–J) In situ hybridization using a *hppy* probe in third instar discs of wild type (I) and *sal^EPv^-Gal4/EP-704* (J) and genotypes. The gene is over-expressed in the *sal^EPv^* domain in *sal^EPv^-Gal4/EP-704* discs, and in all wing disc cells in normal third instar discs. (K–N) Expression and subcellular localization of MAP4K3 in different genetic backgrounds. The protein is localized in the cytoplasm of all wing cells (red signal in K) in wild type discs, it is strongly reduced in discs expressing interference RNA directed against *hppy* (*en-Gal4 UAS-GFP/UAS-ihppy*, L–M), and it is present at higher than normal levels in *nub-Gal4 EP-704/+* wing discs (N). M is the red channel of L, showing the expression of MAP4K3. The basal membrane is labelled in green in K by *vkg-GFP* protein trap (green). N is at double magnification than K-M, and shows the boundary between cells expressing and non-expressing *nub-Gal4*.

Combinations between the P-GS insertion *EP-704* and a variety of Gal4 drivers expressed in the wing disc result in a very consistent reduction of wing size (Fig. 1A–B and Fig. S1). In general, the patterns of veins and sensory organs remain normal in these wings. Phenotypes in which the wing has a normal pattern but is smaller in size are typical of reduced Insulin/Tor signalling [7]–[11], and therefore the gene affected by the *EP-704* insertion is a good candidate to interfere with this signalling pathway. *EP-704* maps within the 5′UTR of *hppy* transcripts (Fig. 1H and Fig. S1), and combinations between *EP-704* and Gal4 lines show increased levels of *hppy* mRNA (Fig. 1J and data not shown). To confirm that *hppy* is responsible for the wing size reductions displayed by *EP-704/Gal4* flies, we over-expressed *hppy* in different *UAS-hppy/Gal4* combinations. These flies show identical phenotypes compared with the corresponding *EP-704*/*Gal4* combinations (Fig. 1D and Fig. S1). Furthermore, the phenotype of *EP-704/Gal4* flies is suppressed when interference RNA directed against *hppy* is expressed in this background (*nub-Gal4 EP-704/UAS-ihppy*; Fig. 1G), confirming that the increased expression of this gene in *EP-704/Gal4* flies is causing the reduction in wing size. MAP4K3 has a Ser/Thr Kinase catalytic domain and a Citron-Homology domain (CNH) [38]. The over-expression of a synthetic protein containing only the kinase domain (Hppy-ΔCN) also results in a strong reduction of wing size (Fig. 1F). In contrast, the over-expression of only the CNH domain (Hppy-ΔK) has no phenotypic consequences (Fig. 1E).

The expression of *hppy* is detected ubiquitously in wild type discs by *in situ* hybridization (Fig. 1I). To determine the subcellular localization of MAP4K3, we generated an antibody against the protein, and found that it is present in the cytoplasm of all wing cells (Fig. 1K). The expression of the protein is strongly reduced in genetic combinations involving the interference RNA directed against *hppy* (*en-Gal4/UAS-ihppy*; Fig. 1L–M), and is detected at higher than normal levels in *UAS-hppy/Gal4* discs (Fig. 1N).

### Loss-of-function analysis

**Figure 2:**
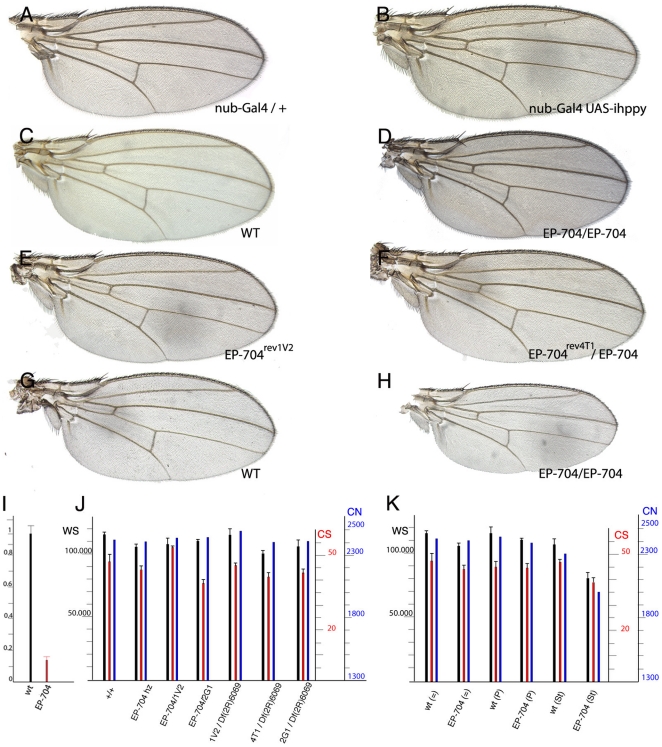
Wing size and cell size phenotypes caused by loss of *CG7097/happyhour* expression. (A) *nub-Gal4/+* control wing. (B) *UAS-dicer*/+;*nub-Gal4/UAS-ihppy*, showing a wild type phenotype. (C) Wild type control wing. (D) *EP-704* homozygous wing. (E) *EP-*704^rev1V2^ in homozygosity. (F) *EP-704* ^rev4T1^ in combination with *EP-704*. (G–H) Control (G) and homozygous EP-704 (H) wings after protein deprivation during the third larval instar. (I) Relative reduction in *happyhour* mRNA levels in EP-704 homozygous larvae (red column). (J) Numeric values of wing size (black), cell size (red) and estimated wing surface cell number (blue) in different heteroallelic combinations of *happyhour* alleles. (K) Numeric values of wing size (WS, black), cell size (CS, red) and estimated wing surface cell number (CN, blue) in wings which larval development occurred in equilibrated media ( = ), in protein-rich media (P) and after protein deprivation (starvation, St).

The strong reduction in MAP4K3 levels observed upon the expression of interference RNA is not associated with any mutant phenotype in the adult wing (Fig. 2A–B). In contrast, the homozygosis of the *EP-704* insertion, although viable, results in smaller than wild type wings, due to a weak reduction in cell size (Table S1 and Fig. 2C–D and 2J). In these flies we detected a reduction of 85% in mRNA levels (Fig. 2I), indicating that the *EP-704* insertion strongly reduces the transcription of *hppy*. We also observed in these flies a developmental delay during the larval stages of approximately 2 days, similar to the delay caused by other *hppy* mutant alleles [36]. In order to generate stronger loss-of-function conditions for *hppy*, we induced by ENU treatment of *EP-704* chromosomes three revertants (*EP-704^rev^*) of the over-expression phenotype (see methods). The molecular lesions of these revertants were identified by sequencing the corresponding coding regions, and they correspond to aminoacid substitutions (*hppy^1V2^*) and to premature stop codons (*hppy^4T1^* and *hppy^2G1^*; see Fig. 1H). Trans-heterozygous combinations between these revertants, or the homozygosity of two of them are viable, and result in wings showing a similar reduction in cell size than the parental *EP-704* chromosome (Table S1 and Fig. 2E–F, 2J). These phenotypes were also similar to those of combinations between deficiencies of the gene and the *EP-704* or *EP-704^rev^* chromosomes (Table S1). Taken together, these results indicate a weak requirement of *hppy* for cellular growth.

Given the reported relationships between MAP4K3 and Tor signalling, we reasoned that the insufficiency for this gene might be more relevant under different nutritional conditions. To analyse this possibility, homozygous *EP-704* larvae were first grown in culture medium with a high level of protein in relation to sugars. In these experiments we found a significant rescue of the growth defect observed in homozygous *EP-704* flies (Fig. 2K and Table S1). Subsequently, we subjected wild type controls and *EP-704* larvae to protein starvation. In this medium homozygous *EP-704* wings are much smaller than controls (Fig. 2G–H, 2K and Table S1), suggesting that the reduction of MAP4K3 affects more significantly cellular growth when the amount of aminoacids is depleted.

### Developmental effects of increased expression of *hppy* in the wing and wing disc

**Figure 3:**
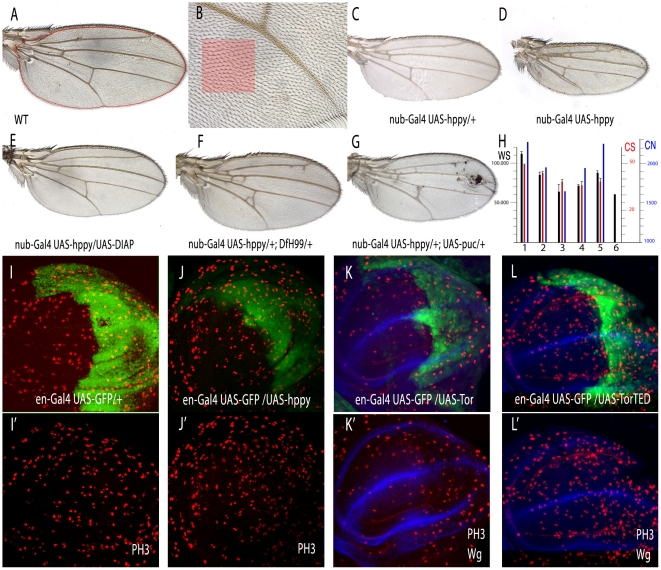
Wing size and cell size phenotypes caused by *hppy* and *Tor* over-expression and their relationships with mitosis. (A–B) Wild type control wings at 5× and 20× magnifications, showing the regions used to measure wing area (A, red line) and cell density (light red square in B). (C–D) Wing phenotypes caused by different levels of *hppy* over-expression in *nub-Gal4 UAS-hppy* /+ (C) and *nub-Gal4 UAS-hppy* /*nub-Gal4 UAS-hppy* (D) wings. (E–G) Wing phenotypes of genetic combinations in which cell death is suppressed or reduced in the background of *hppy* over-expression. (E) *nub-Gal4 UAS-hppy* /UAS-DIAP; (F) *nub-Gal4 UAS-hppy* /+; *Df(3L)H99*/+; (G) *nub-Gal4 UAS-hppy* /+; *UAS-puc/+*. (H) Wing measures of the genotypes showed in A–G. Wing size is shown in the black columns (WS), cell number in the red ones (CS) and estimated cell number in the blue ones (CN). Numbers 1–6 correspond to the following genotypes: *nub-Gal4/+* (1), *nub-Gal4 UAS-hppy /+* (2); *nub-Gal4 UAS-hppy/nub-Gal4 UAS-hppy* (3); *nub-Gal4 UAS-hppy/UAS-DIAP* (4); *nub-Gal4 UAS-hppy/+; DfH99/+* (5) and *nub-Gal4 UAS-hppy /+ UAS-puc/+* (6). (I–L) Expression of PH3 (red) in wild type wing discs (I–I'), *en-Gal4 UAS-GFP/UAS-hppy* (J–J'), *en-Gal4 UAS-GFP/UAS-Tor* (K–K') and *en-Gal4 UAS-GFP/UAS-Tor^TED^* (L–L'). The expression of GFP is in green. The corresponding red and blue (showing Wingless expression) channels are shown below (I'–L').

**Figure 4:**
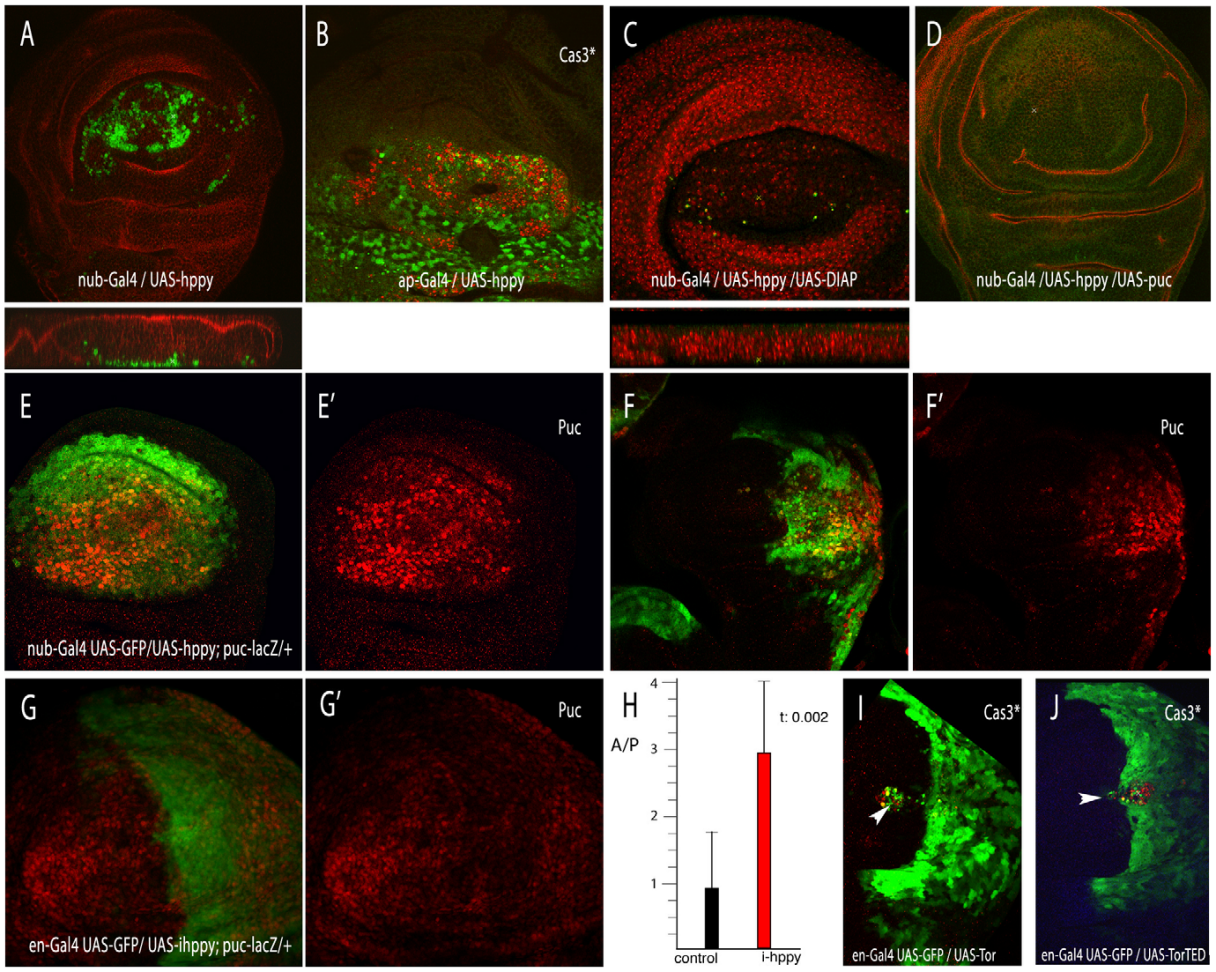
Cell death phenotypes and JNK activation caused by *hppy* and *Tor*. (A–B) Expression of activated Cas3 (green) and FasIII (red) in *nub-Gal4 UAS-hppy*/+ (A) and *ap-Gal4 UAS-hppy*/+ (B) third instar wing discs. (C–D) The presence of apoptotic cells is suppressed when DIAP (C) or Puc (D) are over-expressed with *hppy*. Below A and C are the corresponding Z sections of the discs. In C the nuclei are labelled by Topro (red). (E) Expression of βGal (red) and GFP (green) in *nub-Gal4 UAS-GFP/UAS-hppy; puc-lacZ/+*. (F) Expression of βGal (red) and GFP (green) in *en-Gal4 UAS-GFP/UAS-hppy; puc-lacZ/+*. In both cases there is a strong induction of *puc* expression in the domain of *hppy* over-expression (red channels in E' and F'). (G–G') Expression of *puc-lacZ* (red) in *en-gal4 UAS-GFP/UAS-ihppy; puc-lacZ/+* third instar disc dissected 8 hours after irradiation. The image is a projection of 12 focal planes through the wing disc epithelium. G' shows the corresponding red channel. (H) Ratio between the intensity of the red signal (*puc-lacZ* expression) between the anterior and posterior compartments of *en-Gal4 UAS-GFP/puc-lacZ* (control, black column) and *en-Gal4 UAS-GFP/puc-lacZ; UAS-ihppy/+* (i-happy, red column). The value of the t-student test is also indicated (t: 0.002). (I–J) Induction of cell death in *en-Gal4 UAS-GFP/UAS-Tor* (I) and *en-Gal4 UAS-GFP/ UAS-Tor^TED^* (J) wing discs. The expression of activated Cas3 is in red and GFP in green. The arrowhead points to the places where activated Cas3 is detected.

Due to the weak phenotype of reduced *hppy* expression, we focussed our analysis to the consequences of its over-expression. To evaluate the developmental basis of the *hppy* over-expression phenotype, we measured wing size and cell density in wings expressing different levels of the gene. Wing size was measure in pixels in the area shown in Fig. 3A, and cell size/density was calculated as the number of trichomes (each wing cell differentiates one trichome) in a standard region located in the posterior compartment (red square in Fig. 3B). Increasing the level of MAP4K3 results in progressively smaller wings and reduced cell sizes, with minor vein pattern defects consisting in the formation of extra-vein material (Fig. 3C–D and Table S1). The reduction in the number of cells in wings over-expressing *hppy* varies from 15% to 28% in *nub-Gal4 UAS-hppy* /+ heterozygous compared to *nub-Gal4 UAS-CG7097* homozygous wings (Fig. 3H and Table S1). In this manner, the smaller size of the wing is caused by both a lower than normal number of cells and by a reduction in cell size. The reduction in the number of cells could be due to lower cell proliferation or to increased cell death. To analyse the relative contribution of these processes to the *hppy* over-expression phenotype, we first visualized in *en-Gal4/UAS-hppy* discs the number of cells in mitosis (Phospho-Histone 3, PH3). In these experiments, we over-expressed MAP4K3 only in the posterior compartment, and calculated the mitotic index as the media of the ratios between PH3 positive cells in each compartment and compartment size in 14 wing discs. We found a significant increase in the fraction of mitotic cells in the posterior compartment of *en-Gal4/UAS-hppy* wing discs (Fig. 3I–J). Thus the ratio of the posterior and anterior mitotic indexes in *en-Gal4/UAS-hppy; UAS-GFP/+* discs was 1.8 (sd. 0.26), whereas in *en-Gal4 UAS-GFP/+* controls this value was 1.4 (sd. 0.07). A similar change was also observed in wing discs over-expressing either wild type Tor (Fig. 3K) or the Tor dominant negative form Tor^TED^ (Fig. 3L; P/A ratio is 2.07 and sd. 0.4). The augmented number of mitosis was accompanied by a robust cell-autonomous apoptotic response in discs over-expressing MAP4K3, visualised as an accumulation of cells located close to the basal membrane and expressing activated Caspase3 (Fig. 4A–B). These cells also express *puckered*, a reporter of JNK activity, in *nub-Gal4 UAS-hppy /puc-lacZ* discs (Fig. 4E–F). We suggest that increased levels of MAP4K3 induce apoptosis, likely through JNK activation, and that the surviving cells proliferate more than normal due to compensative proliferation [43]. In fact, when apoptosis is suppressed (Fig. 4C–D), the excess of PH3 positive cells is corrected to near normal values (1.27 P/A index and sd. 0.23). Interestingly, a reduction of *hppy* levels reduces JNK signalling cell autonomously in response to irradiation (Fig. 4G–H), suggesting that MAP4K3 does play a physiological role in the activation of this pathway. This function of MAP4K3 appears to be independent of Tor signalling, because higher than normal levels of Tor and expression of Tor^TED^ only induce very low levels of apoptosis when the miss-expression occurs in one wing compartment (Fig. 4I–J and data not shown).

We also study the contribution of cell death to the phenotype of increased *hppy* expression by suppressing cell death in the *nub-Gal4 UAS-hppy*/+ background. We used the heterozygosis of the *H99* deficiency, which removes the pro-apoptotic genes *reaper*, *hid* and *grim*, as well as the over-expression of dIAP, P35 and Puckered (puc), which are known to suppress cell death acting at different levels of the apoptotic pathway [43]–[46]. In the case of combinations involving *H99*/+ and *dIAP* we find that the reduction of cell death does not significantly rescue the wing size phenotype of MAP4K3 over-expression (Fig. 3E–F and H). When cell death is prevented using the ectopic expression of *puc*, wing size reduction is slightly stronger, and many cells differentiate between the dorsal and ventral wing layers (Fig. 3G–H). Cell size is not affected by the suppression of cell death, and remains similar to that caused by increased expression of MAP4K3 (Fig. 3H). We confirmed that *DfH99*, DIAP and Puc over-expression efficiently suppressed the expression of activated Cas3 in *nub-Gal4 UAS-CG7097*/+ wing discs (Fig. 4C–D and data not shown).

### The effects of MAP4K3 over-expression are similar to those caused by modifications in the TOR and InR signalling pathways

**Figure 5:**
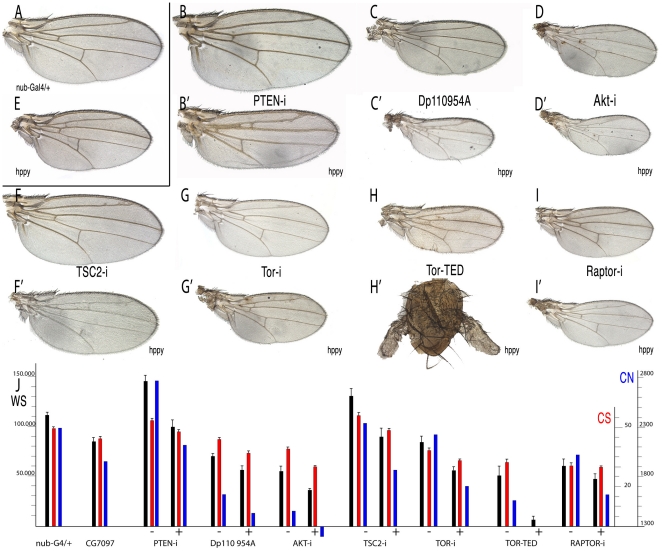
Wing phenotypes of genetic combinations between *hppy* and modifications in InR/Tor activity. (A–I') Representative wings of genetic combinations involving *nub-Gal4* and different UAS constructs affecting InR/TORC1 components alone (B–I) or in combination with UAS-hppy (B'–I'). (A) *nub-Gal4/+* control wing. (B–B') *nub-Gal4/UAS-iPten* (B) and *nub-Gal4/UAS-iPten/UAS-hppy* (B'). (C–C') *nub-Gal4/UAS-Dp110* ^D*954A*^ (C) and *nub-Gal4/UAS-Dp110* ^D*954A*^ *UAS-hppy* (C'). (D–D') *nub-Gal4/UAS-iAkt* (D) and *nub-Gal4/UAS-iAkt/&UAS-hppy* (D'). (E) *nub-Gal4/UAS-hppy* control wing. (F–F') *nub-Gal4/UAS-iTsc2* (F) and *nub-Gal4/UAS-iTsc2/UAS-hppy* (F'). (G–G') *nub-Gal4/UAS-iTor* (G) and *nub-Gal4/UAS-iTor/UAS-hppy* (G'). (H–H') *nub-Gal4/UAS-Tor^TED^* (H) and *nub-Gal4/UAS-Tor^TED^/UAS-hppy* (H'). (I–I') *nub-Gal4/UAS-iRaptor* (I) and *nub-Gal4/UAS-iRaptor/UAS-hppy* (I'). (J) Quantification of wing phenotypes showing the mean values and standard deviation of wing size (WS, black), cell size (CS, red) and estimated cell number (CN, blue) for each combination analysed. The “+” and “−“ signs below each group of three values indicated the data for combinations of *nub-Gal4* with the mentioned UAS line (−) and the same combination including *UAS-hppy* (+).

**Figure 6:**
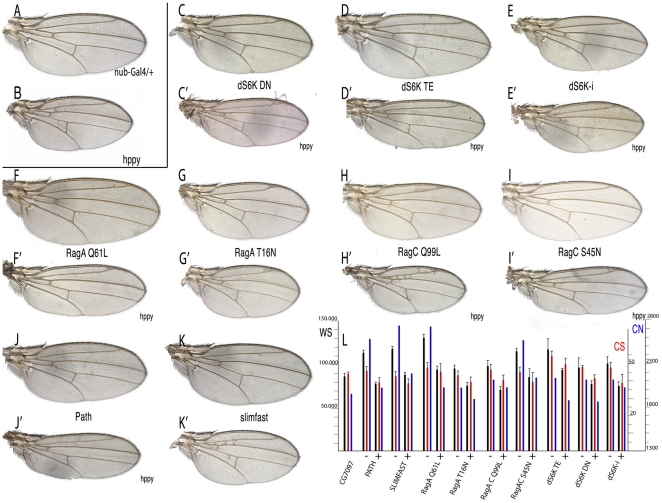
Wing phenotypes of genetic combinations between *hppy* and modifications in InR/Tor activity. (A) *nub-Gal4/+* control wing. (B) *nub-Gal4/UAS-hppy* control wing. (C-K') Representative wings of genetic combinations involving *nub-Gal4* and different UAS constructs affecting InR/TORC1 components, either alone (C–K), or in combination with *UAS-hppy* (C'–K'). (C–C') *nub-Gal4*/*UAS-dS6K^DN^* (C) and *nub-Gal4*/*UAS-dS6K^DN^*/UAS-*hppy* (C'). (D–D') *nub-Gal4*/*UAS-dS6K^TE^* (D) and *nub-Gal4*/*UAS-dS6K^TE^/UAS-hppy* (D'). (E–E') *nub-Gal4/ UAS-idS6K* (E) and *nub-Gal4/* (E'). (F–F') *nub-Gal4*/*UAS-RagA^Q61L^* (F) and *nub-Gal4*/*UAS-RagA^Q61L^/UAS-hppy* (F'). (G–G') *nub-Gal4*/*UAS-RagA^T16N^* (G) and *nub-Gal4 UAS-hppy*/*UAS-RagA^T16N^* (G'). (H–H') *nub-Gal4*/*UAS-RagC^Q99L^* (H) and *nub-Gal4*/*UAS-RagC^Q99L^/UAS-hppy* (H'). (I–I') *nub-Gal4*/*UAS-RagC^S45N^* (I) and *nub-Gal4/ UAS-RagC^S45N^*/*UAS-hppy* (I'). (J–J') *nub-Gal4/UAS-path* (J) and *nub-Gal4/UAS-path*/*UAS-hppy*/ (J'). (K–K') *nub-Gal4/UAS-slimfast* (K) and *UAS-slimfast/UAS-hppy* (K'). (L) Quantification of wing phenotypes showing the mean values and standard deviation of wing size (WS, black), cell size (CS, red) and estimated cell number (CN, blue) for each combination analysed. The “+” and “−“ signs below each group of three values indicated the data for combinations of *nub-Gal4* with the mentioned UAS line (−) and the same combination including *UAS-hppy* (+).

The reduction in cell size observed when MAP4K3 is over-expressed is similar to the effect caused by loss of InR/Tor activity [7]–[11]. To evaluate directly the consequences of loss of InR/Tor signalling in the wing disc, we expressed several interference RNAs or modified versions of different components of the InR/Tor pathway in this tissue (Fig. 5, 6 and Table S1). We found changes in wing size and cell size consistent with the known requirements of this pathway during imaginal development [7]–[11]. Thus, loss of Akt (Fig. 5D) and Tor (Fig. 5G) results in a 27–30% increase in cell density, and expression of dominant negative versions of Dp110 (Fig. 5C), RagA (Fig. 6G) and dS6K (Fig. 6C) caused similar but weaker effects on cell size (7–14% increase in cell density; Fig. 6L and Table S1). The corresponding wings are smaller than normal in size, and these reductions are in part due to a lower than normal number of cells (Fig. 5J, Fig. 6L and Table S1). When the activity of the pathway is increased by expressing activated forms of Akt (Table S1), RagA (Fig. 6F) and dS6K (Fig. 6D), or by a reduction in the negative modulators Pten (Fig. 5B) and TSC2 (Fig. 5F), cell size is only moderately affected and wing size is generally larger than normal (ranging from 5% in the case of activated dS6K to 32% in the case of loss of Pten; Fig. 5J, Fig. 6L and Table S1). The estimated number of cells in these combinations is also higher than normal, particularly in the cases of activated RagA and loss of Pten (20% more cells than normal; Fig. 5J, Fig. 6L and Table S1). In this manner, the effects caused by over-expression of MAP4K3 are similar to those observed when InR/Tor activity is reduced, with the important difference that a reduction in InR/Tor signalling does not lead to significant cell death in most of the combinations analysed (Fig. 4 and data not shown).

### MAP4K3 and several components of the Tor pathway display genetic interactions

To identify a functional relationship between TORC1 and MAP4K3, we first expressed interference *hppy* RNA in genetic backgrounds with modified InR/TORC1 activity (RagA^wt^, RagA^Q65L^, RagA^T16N^, RagC^S45N^, RagC^Q99L^, Tor^TED^, Raptor-i, dS6K^TE^, dS6K-i, Pten-i, Akt-exel, Tsc1-i). Except in the case of Tor^TED^ (see below), the resulting phenotypes were in all cases additive (data not shown), and therefore we concentrated in combinations involving over-expression of *hppy* in InR/TORC1 mutant backgrounds. Three key components of InR/Tor signalling upstream of the TORC1 complex are the phosphatase Pten, the Ser/Thr kinase Akt/PKB and the TSC1/2 complex. Loss of PTEN leads to increased levels of PI3P, a positive regulator of Akt, and Akt activity is required to phosphorylate and reduce the levels of TSC1/2 [17]–[19]. We find that in combinations between loss of PTEN and over-expression of *hppy* (*nub-Gal4/UAS-hppy; UAS-iPTEN/+*), the cell size phenotype is intermediate, and the wing size is more similar to over-expression of *hppy* alone (Fig. 5B', compare with 5B and 5E). The number of cells in these combinations is reduced to a similar extent to that of wings over-expressing *hppy* (Fig. 5J). Similarly, over-expression of *hppy* rescues the phenotype of TSC1/2 loss (*nub-Gal4/UAS-hppy; UAS-iTSC2/+*, Fig. 5F', compare with 5F and 5E), resulting in wings similar to those over-expressing *hppy* (Fig. 5J). These results suggest that higher than normal levels of MAP4K3 block or antagonise the effects of high level of InR signalling generated downstream of Akt and TSC1/2. As expected, a reduction in the level of Akt in the background of *hppy* over-expression (*nub-Gal4 UAS-hppy /UAS-iAkt*) results in a strong synergistic phenotype of reduced cell size and number (Fig. 5D', compare with 5D and 5E). A similar result is also observed when *hppy* is over-expressed in wings expressing the Dp110 dominant negative form (*nub-Gal4/UAS-hppy; UAS-Dp110^D945A^/+*, Fig. 5C', compare with 5C and 5E).

We next tested the possible genetic interactions between MAP4K3 and several components of the TORC1 complex, including Raptor, RagA, RagC and Tor. In the case of Tor, we observed strong synergistic interactions when the level of MAP4K3 is increased in a background of cells with lower amount of Tor (*nub-Gal4 UAS-hppy /UAS-iTor*; Fig. 5G', compare with 5G and 5E) or expressing the dominant negative form Tor^TED^ (*nub-Gal4 UAS-hppy /UAS-Tor^TED^*; Fig. 5H', compare with 5H and 5E). These effects are manifested in the reduction of cell and wing size, and consequently also in the total number of cells estimated for these wings (Fig. 5J). Similar results were observed in the case of combinations with Raptor, a key member of the TORC1 complex (*nub-Gal4 UAS-hppy /UAS-iraptor*; Fig. 5I', compare with Fig. 5I and 5E). Combinations between activated or dominant negative forms of RagA and over-expression of *hppy* were particularly informative. Thus, the over-expression of *hppy* cancels the effect of activated RagA (*nub-Gal4 UAS-hppy /UAS-iRagA^Q61L^*; Fig. 6F', compare with 6F and 6B, and Fig. 6L), and enhances the phenotype of a dominant negative RagA form (*nub-Gal4 UAS-hppy /UAS-RagA^T16N^*; Fig. 6G', compare with 6G and 6B). Interactions with RagC were, as expected opposite to those with RagA. Thus, activated RagC, which is expected to reduce the activity of the TORC1 complex, further aggravates the reduction in cell size caused by excess of hppy (Fig. 6H', compare with 6H and 6B). These results suggest that MAP4K3 acts downstream of RagA, and that the effects of its over-expression are extremely sensitive to the levels of RagC, Tor and Raptor.

In addition to InR signalling mediated by Akt/TSC1-2/Rheb, TORC1 activity is also regulated by the level of intracellular aminoacids [30]–[31]. Although this pathway is less well characterised, at least two amino acid transporters, encoded by *pathetic* (*path*) [31] and *slimfast* [30] have being suggested to mediate this branch of TORC1 regulation. The over-expression of either *path* or *slimfast* affects mostly cell size without changing the final size of the wing, and consequently these wings are formed by a larger than normal estimated number of cells (Fig. 6J and 6K, respectively and Table S1). When we over-expressed *hppy* in these backgrounds, the resulting wings have cell sizes, cell numbers and wing size similar to *hppy* over-expressing wings (Fig. 6J' and K', compare with 6J and 6K and with Fig. 6B). Finally, in combinations involving different forms of one of the downstream components of the pathway, dS6K, and over-expression of *hppy*, we found that expression of constitutively activated dS6K diminishes the cell size reduction caused by MAP4K3 (*nub-Gal4 UAS-hppy /UAS-dS6K^TE^*; Fig. 6D', compare with 6D and Fig. 6L), and that dominant negative dS6K or interference RNA against its transcript causes a moderate synergistic phenotype of reduced cell size (*nub-Gal4 UAS-hppy /UAS-dS6K^DN^*; Fig. 6C', compare with 6C and *nub-Gal4 UAS-hppy /UAS-idS6K*; Fig. 6E', compare with 6E and 6B). Taken together, these results suggests that MAP4K3 acts downstream of the aminoacid transporters, and upstream of dS6K activity. The numerical values, standard deviations and t-test values of the wings discussed in this and the previous section are presented in Table S1.

### MAP4K3 interacts with the TOR complex

**Figure 7:**
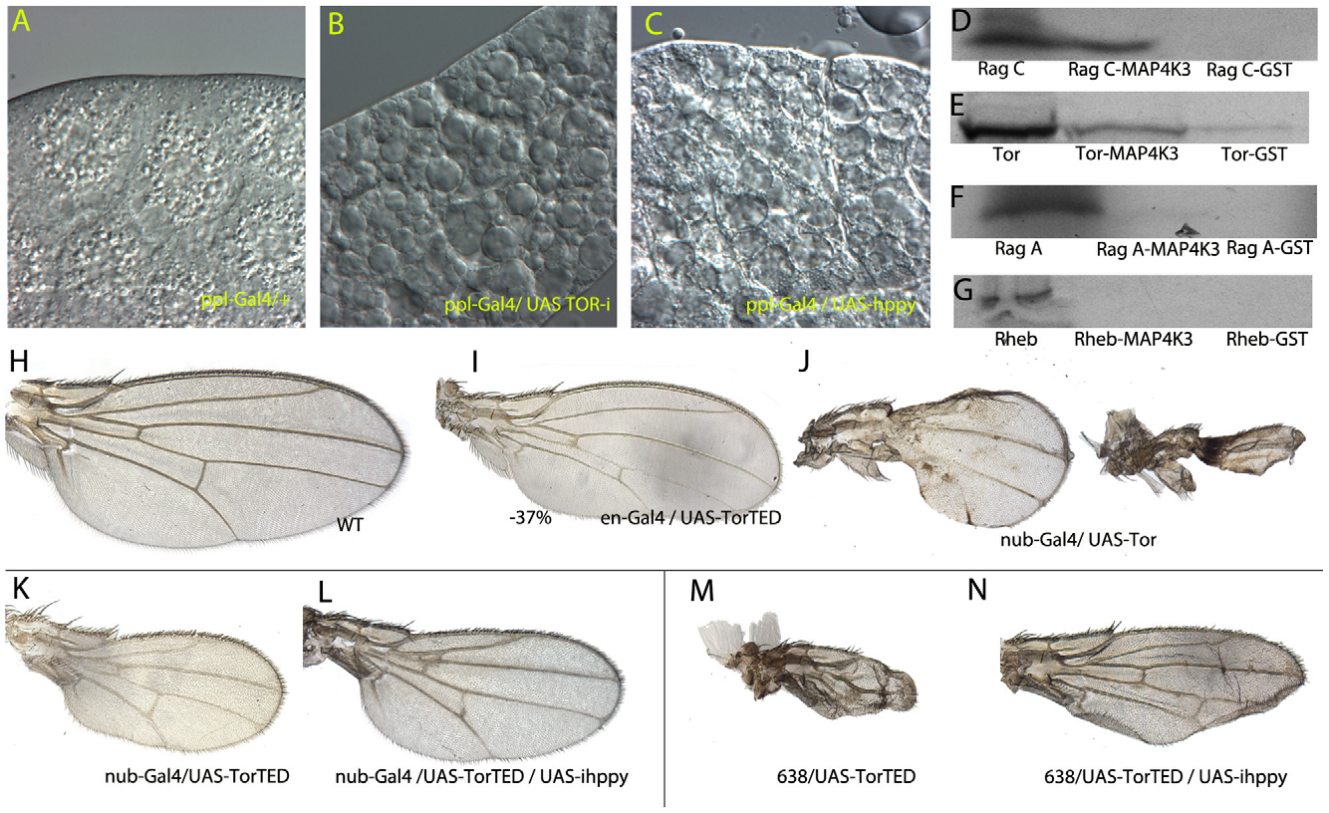
Relationships between CG7097 and the TORC1 complex. (A–C) Fat body phenotypes in control larvae (*ppl-Gal4/+*; A), and after loss of Tor expression (*ppl-Gal4/+; UAS-iTor/+*; B) or MAP4K3 over-expression (*ppl-Gal4/UAS-hppy*; C). (H–J) Control wing (H), and wings corresponding to over-expression of *Tor^TED^* in the posterior compartment (*en-Gal4/UAS-Tor^TED^*; I) and Tor in the entire wing blade and hinge (*nub-Gal4/UAS-Tor*; J). Note the variable reduction in wing size. (D–G) Pull-down analysis of GST-MAP4K3 fusion protein and in vitro translated RagC (D), Tor (E), RagA (F) and Rheb (G). In each autoradiography the left lane corresponds to the in vitro translation reaction, the central lane to the inmunoprecipitation with GST-MAP4K3, and the right late to inmunoprecipiation with GST. Note that RagC and GST-MAP4K3 interact very efficiently. Tor and GST-MAP4K3 also interact but to a lesser extent, and in this last case we also detected some interaction between Tor and GST. The interaction between RagA and GST-MAP4K3 is very weak, and we could not detect any interaction between Rheb and GST-MAP4K3. (K–N) Reduction of the *Tor^TED^* phenotype by the loss of *hppy* expression. (K–L) *nub-Gal4*/ *UAS-Tor^TED^* (K) and *nub-Gal4*/ *UAS-Tor^TED^* /*UAS-ihppy* (L). (M–N) *638-Gal4*/+; *UAS-Tor^TED^* (M) and *638-Gal4*/+; *UAS-Tor^TED^*/*UAS-ihppy* (N).

The phenotype of *hppy* loss and over-expression and the results of genetic combinations with different members of the InR/Tor pathways suggest that MAP4K3 is functionally related to TORC1 activity. To confirm the link between MAP4K3 and TORC1, we explored whether *hppy* affects other developmental processes regulated by TORC1, and analysed the possibility of direct interactions between MAP4K3 and several components of the TORC1 complex in pull-down experiments. We looked at the development of the fat body, the fly equivalent to the vertebrate liver, where loss of TORC1 signalling causes the fusion of lipid vesicles (47 and Fig. 7A–B), an effect also caused by aminoacid deprivation [30], [47]. The over-expression of *hppy* in this tissue also causes this effect (Fig. 7C, compare with 7A), suggesting that *hppy* over-expression interferes with TORC1 signalling in different developmental contexts.

The phenotype of *hppy* over-expression is therefore in part related to insufficiency of TORC1, and this could be interpreted as the consequence of a normal requirement of *hppy* to regulate negatively TORC1 activity. This conclusion, however, is in contrast with the phenotype of *hppy* hypomorphs [36], which suggested that MAP4K3 is required to activate the TORC1 complex [35]. Several lines of evidence indicate that the levels of Tor itself are critical for the correct activity of TORC1. In this manner, the over-expression of Tor causes a strong and variable phenotype of wing size reduction that is reminiscent of loss of TORC1 function (Fig. 7J and 48). Because of the strength and variability of this phenotype, we could not analyse the effects of changing the levels of MAP4K3 in this genetic background. Over-expression of the dominant negative form of Tor, Tor^TED^, also results in a severe reduction of cell size and wing size (Fig. 7I, K and M, compare with 7H) which strength depends on the Gal4 driver used (compare Fig. 7K with Fig. 7M). Interestingly, these genetic backgrounds are very sensitive to the loss of *hppy* expression, because the lowering *hppy* levels significantly rescues the reduced wing size defect cause by Tor^TED^ over-expression (Fig. 7L, compare with 7K and Fig. 7N, compare with 7M). As TORC1 function is extremely sensitive to the level of its components, a possible mechanism to explain the phenotype of *hppy* over-expression consistent with these observations is through changes in the correct balance between members of the complex. We explored the possibility of direct interactions between MAP4K3 and some of TORC1 components in pull-down experiments using MAP4K3 and in vitro translated RagA, RagC, Tor and Rheb. We found that MAP4K3 can interact directly and efficiently with RagC (Fig. 7D), and less so with RagA and Tor (Fig. 7F and E, respectively). Similar results concerning RagA and RagC were found in inmunoprecipitation studies carried out in Drosophila culture cells [36]. We could not detect direct interactions between Rheb and MAP4K3 (Fig. 7G), suggesting that the over-expression of MAP4K3 might disrupt the formation of functional TORC1 complexes downstream of Rheb.

## Discussion

We have characterised the consequences of changing the amount of MAP4K3, encoded by *hppy*, in the development of the wing disc, focussing on its relationships with the TORC1 signalling pathway. Previous data suggested that MAP4K3 might be related with a variety of signalling pathways, including EGFR [37], ImD [40], JNK [41] and TOR [35], [36]. For these reasons, we used the advantages of the wing model to analyse *hppy*, as in this system changes in the level of signalling by a variety of pathways lead to pathway-specific phenotypes. A reduction of *hppy* expression in the wing, using interference RNA or loss-of-function alleles, did not uncover a critical requirement of the gene for embryonic or larval viability. In *hppy* mutant wings we only found a weak reduction in wing size and cell size, which is compatible with a moderate reduction of TORC1 activity. It has been recently reported that the developmental delay caused by protein starvation is similar in wild type and *hppy* mutant larvae, suggesting that MAP4K3 is required in vivo to activate TOR and promote growth mostly when amino acid conditions are rich [36]. In contract, we found a significant requirement for the gene when *hppy* mutant larvae grow under starvation conditions. Thus, these flies still develop smaller wings than controls, indicating a functional requirement of *hppy* when the availability of proteins is reduced. This difference could be due to the parameters measured (developmental delay vs. cell and wing size) or to the remnants of *hppy* function in the alleles used in each experiment.

We also found that, loss of *hppy* does not affect cell viability or JNK signalling, but that in a *hppy* loss-of-function genetic background the activation of JNK signalling in response to irradiation is reduced. Thus, the function of *hppy* might become significant mostly when the organism is challenged by stress signals induced for example by irradiation, indicating a role for the gene in the modulation of JNK signalling in vivo.

The increase in *happyhour* expression does have more dramatic consequences that its loss, causing a severe reduction in the size of the wing independently of environmental conditions. Wing size reduction is associated with both apoptosis and a smaller than normal cell size. The overall morphology and pattern of these wings is normal, with only a weak phenotype of extra-veins in the strongest combinations. Cell death induction and reduced cell size are the diagnostic phenotypes of increased JNK and reduced InR/Tor signalling, respectively. The same processes are affected by loss of MAP4K3 expression in the wing, and therefore, from this analysis we conclude that MAP4K3 has the potential to activate cell death through the JNK signalling pathway, and also that it can interfere with some component/s of the InR/Tor cascade. The effects of loss- and gain of MAP4K3 on JNK activity are opposite, which is expected from a protein with kinase activity. In contrast both loss and gain of MAP4K3 seem to reduce the function of TORC1. It is likely that in this case MAP4K3 acts as part of a protein complex that can be made non-functional by changes in the stechiometry of its components. What seems clear is that the effects of MAP4K3 on JNK and TORC1 are exerted through independent mechanisms, because the contribution of cell death to the wing phenotype of MAP4K3 over-expression is very modest, and Tor reductions only lead to cell death when cells with different levels of Tor activity are confronted.

The phenotype of MAP4K3 over-expression is very sensitive to changes in the levels or activity of several members of the InR/Tor pathway. Thus, strong synergistic interactions were observed when *Akt*, *raptor* and *Tor* are reduced in the background of MAP4K3 over-expression, and the presence of the dominant-negative form Tor^TED^ in this background entirely eliminates the wing. Conversely, loss of *hppy* expression rescues the effects of Tor^TED^ expression. These results suggest that MAP4K3 could act at the level of TORC1. This possibility is compatible with the suppression by MAP4K3 over-expression of phenotypes caused by increased levels of InR/Tor signalling generated by lower than normal levels of PTEN and TSC1/2. In addition to our genetic data in the wing, experiments in cell culture with both the fly and human MAP4K3 homologue proteins indicated that MAP4K3 is required to generate maximal activity of TORC1 in response to aminoacids [35]. Therefore, we suggest that although MAP4K3 is normally required to promote TORC1 signalling, when the protein is over-expressed modifies the balance between TORC1 components required for its normal function in vivo. This effect appears to depend exclusively on the kinase domain of MAP4K3, because the over-expression of this domain causes a strong reduction in wing and cell size. We have shown that MAP4K3 can interact with RagA, RagC and Tor in pull-down experiments in vitro, and therefore we speculate that the excess of MAP4K3 alters the phosphorylation levels of TORC1 components and this leads to the assembly of inactive complexes. A similar mechanism might explain the dominant-negative effect of Tor, as it was suggested that Tor over-expression leads to the sequestering of TORC1 components in non-functional complexes [48]. In summary, we suggest that MAP4K3 normally potentiate TORC1 and JNK functions in response to environmental challenges, without being strictly required to generate some levels of TORC1 or JNK activity, and that MAP4K3 hyper-activity leads to high levels of JNK signalling and to reduced TORC1 function, in this case due to the formation of inactive TORC1 complexes.

## Methods

### Genetic strains

We used the EP line *EP-704* [5], the Gal4 lines *act-Gal4*, *ap-Gal4, 638-Gal4*, *nub-Gal4, ap-gal4, dpp-Gal4, omb-gal4, hh-Gal4, en-Gal4, sal^EPv^-Gal4* [6] and *ppl-Gal4* [49], the UAS-lines *UAS-GFP* [50], *UAS-diap, UAS-puc* [51], *UAS-Tor^WT^* and *UAS-Tor^TED^* [48], *UAS-Dp110^D954A^* [52], *UAS-RagA^Q61L^*, *UAS-RagA^T16N^*, *UAS-RagA* and *UAS-RagC* [32], *UAS-dS6K^TE^* [53], *UAS-dS6K^DN^*, *UAS-pth* [31], *UAS-AKT^exel^* and *UAS-slif* [30]. We also used *Df(2R)Exel6069* as a deletion of *hppy*. The expression of *638-Gal4* and *nub-Gal4* is restricted to the wing pouch and wing blade plus hinge, respectively, since the second larval instar. The expression of *hh-Gal4* and *en-Gal4* is restricted to the posterior compartment of all imaginal discs, the expression of *act-Gal4* occurs in all imaginal cells and the expression of *ppl-Gal4* occurs in the fat body. We also used the following UAS lines to express interference RNA: *UAS-iTor* (ID 5092-R2 DGRC), *UAS-iraptor* (ID 4320R-2 DGRC), UAS-idS6K (ID 10539-R3 DGRC), *UAS-iAkt* (ID2902 VDRC), *UAS-iPTEN* (ID 35731 VDRC), *UAS-iTSC2* (ID 6313 VDRC) and *UAS-iCG7097* (ID 35166 VDRC). Unless otherwise stated, crosses were done at 25°C. Lines not described in the text can be found in FlyBase [54].

### Generation of *happyhour* loss-of-function alleles

Isogenic *EP-704* males were treated with 0,4 mM Ethyl Nitrous Urea (ENU) overnight is a Sucrose solution. Treated males were crossed with *CyO/act-Gal4* females. Viable individual males of EP-704*/ act-Gal4 genotype were selected to establish stocks carrying mutations in *hppy* that suppress the over-expression phenotype of *EP-704/Gal4* combinations. *EP-704/CyO act-Gal4* flies are pupal lethal.

### Mapping of *happyhour* loss-of-function alleles

The three revertants we isolated were crossed with *Df(2R)tExel6069/CyO* (a deficiency including the CG7097 genomic region). We extracted genomic DNA from EP704*/Df(2R)Exel6069 and used it as a template to amplify the coding genomic fragments of CG7097 by PCR. We used the following primer pairs:

Exon 1: TGCATCTCGATCCACAAAAG and AGTTTTTGCCTTTCCCTTCC

Exon 2: AAAGCCACCACATAATCTCCA and CCCCGCCTTTAAATCTGACT

Exons 3–4: TTAGCTGAGGCAGCAAAACC and TAAAGGCAAAAGGTGCCAAG for Exons 5–6: GTGAAGGTCCATTCCTCGAA and AACCCGTTTCGCAAGTAAGA

Exon 7: CGTTTAATTTGGGCGTTAGGA and TTGCGTGAAAATATGGTGGA

Exon 8: GCTCAACATCTCGGCTCTCT and CATTATTCATGGCCACATCG

Exon 8: ATGCCGACGACGATGAACT and TACCTGAACCCCATTTGATG

Exons 9 and 10: TGATTACCGGCATGAGAACA and CTCCGGGATCTTGTTCATGT

Exon 11: TGGGTACACGATTCTTGCAT and ATGGCGTCATATTCCAGGTC

Exons 12–14: GCTGGAGCTGATCAACATGAA and TGTGTGGTGAACCGGATTAT

The PCR products were cloned in PGem-T-Easy (Promega) and sequenced with T7 and SP6 primers. Two independent PCR reactions were set for each primers pair, and 2 independent clones were sequenced from each PCR. We found a T by A substitution in exon 2 codon TT^*^C of EP704^1V2^ (resulting in a F to Y aminoacid change), a C by T substitution in exon 1 codon C^*^AA of EP704^4T1^ (resulting in a Q to stop codon change) and a C by T substitution in exon 11 codon C^*^AG of EP704^2G1^ (resulting in a Q to stop codon change).

### Starvation conditions

Control and homozygous *EP-704* early third instar larvae were removed from the food, washed in PBS and placed in vials containing paper saturated with H_2_O and sucrose. The imago ecloses after 5–6 days under these conditions.

### Quantitative RT-PCR

Total RNA was prepared from a pool of 20 wild type and 20 *EP-704/EP-704* 3rd instar larvae using the TRIzol reagent RNA protocol following GibcoBRL instructions. Total RNA was used for a first round of reverse transcription using the Gene Amp RNA PCR kit (Applied Biosystems, Foster City, CA). Quantitative Real-time RT-PCR was performed using SYBR® green PCR master mix and the ABI PRISM® 7700 sequence detection system (Applied Biosystems). Primers for real-time RT-PCR were for CG7097 -forward TCC CAA GCA GCA CAA GAA GA- and -reverse CTC TCC AAG GCC ACA ACC TT-, and for RP49 controls –forward CGG ATC GAT ATG CTA AGC TGT– and –reverse CGA CGC ACT CTG TTG TCG-. Quantifications were always normalised using endogenous control *RP49* (ribosomal protein 49).

### Generation of *UAS-hppy, UAS-hppy-ΔCN* and *UAS-hppy-ΔK*

UAS-hppy was constructed cloning the complete RH10407 cDNA (from Berkeley Drosophila Genome Project) into the pUASt vector [54]. The EST RH10407 was used as template for amplification using the forward primer 5′ TATGTCGACGATTTGTGGGCTGCG-3′ and the reverse primer5′-GAAGCGGCCGCTGCACTAAGCTAGAG-3. This fragment was purified (Promega) and cloned into the pUASt vector digested with SalI and NotI restriction enzymes (underlined in the primers). UAS-hppy-ΔCN and UAS-hppy-ΔK were constructed cloning the cDNA RH10407 fragments between 16 bp to 1988 bp and between 1260 bp to 3911 bp, respectively, into the pUAST Drosophila Gateway™ Vector using Invitrogen pENTR™ Directional TOPO® Cloning Kits. The primers used for this purpose were for UAS-hppy-ΔCN 5′ forward 5′-CACCCGTTGACGAAGTGCATGTG-3′ and reverse 5′-TTAGGTGTTGTTCAGCAGATCC-3′; and for UAS-hppy-ΔK forward 5′-CACCATGCCCAACCCGCAGTTCTACTA-3′, reverse 5′-CCCAACAAACCGTATCATCC-3′. Several UAS lines for each construct were established after germ-line transformation following standard procedures.

### Wing and cell size measurements

We analysed at least 10 wings from females of each genotype. Cell size was estimated by counting the number of wing hairs in a dorsal region posterior to the posterior crossvein. Wing size was measured in pixels using the Analyse tool in Adobe Photoshop. The number of cells was calculated using cell density and wing size values.

### Mitotic index

We quantified the number of cells in mitosis, detected by the expression of Phospho-Histone3, in the anterior and posterior wing disc compartments, and the size of the corresponding compartments. The average value of the ratios between the number of mitotic cells and the size of the compartment was used as a compartment mitotic index. The images used to make the quantifications were projections of at least 10 confocal planes including the entire apico-basal height of the wing blade epithelium. We did these measures in at least 10 third instar wing discs of the following genotypes: *en-Gal4 UAS-GFP/UAS-CG7097*, *en-Gal4 UAS-GFP /UAS-Tor*, *en-Gal4 UAS-GFP/UAS-Tor^TED^*, *en-Gal4 UAS-GFP/UAS-CG7097; UAS-diap*/+ and *en-Gal4 UAS-GFP /+.* The expression of GFP was used to label the posterior compartment.

### Generation of anti-MAP4K3 antibody

The anti-MAP4K3 antibody was prepared by immunizing rats with a Poly-Histidine fusion peptide of aminoacids 379-696 of *Drosophila* Hppy (isoform B) after PCR cloning from *hppy* cDNA (*RH10407*) into *pQE31* (Qiagen).

### Visualization of JNK activity after irradiation

Third-instar larvae of *en-Gal4 UAS-GFP/UAS-ihppy; puc-lacZ* and *en-Gal4 UAS-GFP/+; puc-lacZ* genotypes were irradiated in a Philips X-Ray machine to a final dose of 1500R. The imaginal discs were dissected and stained 8 hours after the irradiation. We took Z-projections made from 50 tangential sections and the intensities of the red channel (*puc-lacZ* expression) in the anterior and posterior compartments were measured using ImageJ1.38 software (NIH).

### Immunocytochemistry and in situ hybridization

We used rabbit anti-activated Cas3 (Cell Signalling Technology), anti-Phosphorylated Histone (PH3; Cell Signalling Technology), mouse anti-βGalactosidase (Promega) and TOPRO (Invitrogen). From the Hybridoma bank at Iowa University we used monoclonals anti-Wg and anti-FasIII. Secondary antibodies were from Jackson Immunological Laboratories (used at 1/200 dilution). Imaginal wing discs were dissected, fixed and stained as described in [56]. Confocal images were captured using a LSM510 confocal microscope. In situ hybridization with *hppy* RNA probes was carried out as described in [55]. We used the cDNA RH10407 as a template to synthesise the *hppy* RNA probe.

### Pull-down assays

We made a GST (Gluthatione S-Tranferase) fusion protein of the complete *Drosophila* CG7097 isoform B. The EST *RH10407* was used as template for amplification using the forward primer 5′-GGGGGTCGACGGTTGACGAAGTGCATGTGAAA-3′ and the reverse primer 5′-GGGGGCGGCCGCTCGTGTGGATAATTGCGTGT-3. This fragment was purified (Promega) and cloned into the PGEX-4T-1 vector digested with *Sal*I and *Not*I restriction enzymes (underlined in the primers). Subsequently, the fusion protein was purified in a Gluthatione-agarose column for 2 h at 4°C. Rheb (GH10361), RagA (GH04846), RagC (GH16429) and dTOR (RE49094) proteins where generated from their cDNA vectors (Berkeley Drosophila Genome Project) with the in-vitro translation kit TNT T7 coupled Reticulocyte Lysate Systems (Promega) and radiolabeled with S^35^-Met. The pull-down assay was performed over-night at 4°C using 10 µl in-vitro translation, 50 µl CG7097-GST or GST and 200 µl of pull-down buffer (20 mM Tris-HCl pH 8, 150 mM NaCl, 1 mM EDTA and 0,5%NP40). After centrifugation and washes the proteins were separated in a 10% SDS/PAGE gel and the existence of pull-down proteins was analysed by autoradiography. The levels of GST fusion proteins and GST protein were quantified on 10% SDS-PGE gels with known levels of BSA as reference. After that, the pull-downs were carried out with the same amount of GST fusion proteins.

### Western Blot

Control S2 cells or S2 over-expressing hppy were collected by centrifugation, washed with PBS, re-suspended in 100–200 µl of ice-cold lysis buffer (50 mM Tris-HCl, 150 mM NaCl, 0,5% NP40, 2 mM EGTA, 2 mM EDTA, 10 mM NaF, 0.1 µM orthovanadate, 100 µM PMSF, 1 mM Benzamidine, 16 mU/ml Aprotinin, 5 mM DTT) and incubated for 1–2 h at 4°C. Lysates were clarified by centrifugation.

Whole S2 lysate complexes were resolved by 10% SDS-PGE and proteins transferred to nitrocellulose membranes using a wet-blotting apparatus (BioRad). Hppy protein was detected with anti-Hppy rat serum (1/200). Blots were also revealed with anti-gammaTubulin monoclonal antibody (Sigma) as a loading control. Immunoblots were developed and quantified using IR680 and 800 labelled antibodies (Licor) with the Odyssey Infrared Imaging System (Li-Cor).

### Statistical analysis

Data are expressed as means +/− standard error of the mean (SEM). All data were collected in a Microsoft Excel database (Microsoft Inc.), and figures were created using Adobe Illustrator CS3 (Adobe Systems Inc.) software.

## Supporting Information

Figure S1 Phenotype EP-704 and UAS-hppy combinations with different Gal4 at 25°C. (A) Wild type wing. (B) EP-704/hh-Gal4. hh-Gal4 is expressed in the posterior compartment of the wing disc, and the wing shows a 15% reduction of wing size. (C) UAS-hppy/hh-Gal4 wing, showing the same reduction of wing size than EP-704/hh-Gal4 wings (B). (D) EP-704/ap-Gal4. ap-Gal4 is expressed in the dorsal compartment of the wing disc, resulting in a 34% of reduction of wing size. (E) EP-704/638-Gal4. 638-Gal4 is expressed in the entire wing blade, and the wing size is reduced 28% compared to wild type wings. (F) EP-704/omb-Gal4, omb-Gal4 is expressed in the most of the wing blade, resulting in wings 25% smaller than controls. (G) EP-704/179-Gal4, 179-Gal4 is expressed in the entire wing blade; being excluded from the D/V boundary, and the wings are 36% reduced in size. (H) EP-704/dpp-Gal4, dpp-Gal4 is expressed in a stripe of anterior cells abutting the A/P boundary, and the wings are reduced 13% in size. (I) EP-704/ salEPv-Gal4, salEPv-Gal4 is expressed in the central region of the wing blade (LII-L5 veins), and the wings are 19% reduced in size. (J) Insertion site of the EP-704 element shown in the genomic region (left) and in the graphical map of the gene (right). (K) Western blot showing the CG7097 protein extracted from wild type cells (left lane, control), and from cells over-expressing Hppy (right lane, over-expression). γ-Tubulin is also shown as a loading control at the bottom.
(2.22 MB TIF)

Table S1 Numerical values of average wing size, average number of cells in the square shown in Fig. 2B, average cell size and estimated number of cells in most of the combinations analysed in this work. Standard deviations are shown in brackets. The values of the t-Student test for each pair of values (T) is also shown.
(0.05 MB XLS)
